# Mentoring, Training and Support to Global Health Innovators: A Scoping Review

**DOI:** 10.5539/gjhs.v5n5p162

**Published:** 2013-06-28

**Authors:** Dan-Bi Cho, Donald Cole, Ken Simiyu, Winnie Luong, Vic Neufeld

**Affiliations:** 1 Global Health Division, Dalla Lana School of Public Health, University of Toronto, Toronto, ON, Canada; 2 Institute for Global Health Equity & Innovation & Global Health Division, Dalla Lana School of Public Health, University of Toronto, Toronto, ON, Canada; 3 Canadian Coalition for Global Health Research, Ottawa, ON, Canada; 4 Grand Challenges Canada at the Sandra Rotman Centre, Toronto, ON, Canada; 5 Department of Clinical Epidemiology & Biostatistics, McMaster University, Hamilton, ON, Canada

**Keywords:** developing countries, technological innovations, organizational innovation, mentors, training support

## Abstract

Global health innovators must navigate substantial complexities to successfully develop, implement and sustain global health innovations with impact through application of an Integrated Innovation™ approach. We sought to examine the nature of the literature and evidence around mentoring, training and support of global health innovators. We conducted a scoping review searching eight databases with terms capturing different kinds of innovation and support. Assessment of relevance and mapping was completed by two reviewers, with interpretation by the review team. Twenty-eight relevant papers provided perspectives on fostering global health innovators and innovation. Fifteen included empirical data on supports to global health innovators involving a wide range of innovators. Eight included documentation of outcomes but without designs to determine effectiveness. The diverse mentoring, training and support activities included: business incubators, support organizations and centres for entrepreneurship, technology transfer and intellectual property management, internship programs for business skill development, initiatives to bridge industry and researchers, and platforms for South-led innovation for global health. We propose the cultivation of a pipeline of global health innovators to increase the number of appropriate, sustainable innovations with impact in global health. Further empirical work on how to effectively support global health innovators is needed.

## 1. Introduction

Global public health is being impacted by ongoing innovation in a myriad of ways that may or may not promote health equity (Cozzens & Kaplinsky, 2009), depending on the nature of the innovation's development and its scale-up (McGahan, 2012). Health innovations such as new vaccines, diagnostic devices, product development partnerships and preventive and therapeutic interventions are being rolled out in low- and middle- income countries (LMICs), stimulating growth in their economies (Gardner, Acharya, & Yach, 2007; Yamey, 2012; Morel et al., 2005). Innovation for global health can mean “…to [take] up novel ideas, inventions or processes and [apply] them to achieve improved health and greater health equity” (Matlin, 2008, p. 13). Global health innovation can be facilitated through various approaches such as venture funding for biotechnology, public-private partnerships, and novel models of health service delivery. However, these mechanisms are most often focused on various segments within an innovation cycle – either discovery (involving basic research), development (clinical development), or delivery (ensuring end users receive products) (Matlin, 2008). Other approaches to global health innovation include the work of the Bill and Melinda Gates Foundation, which is focused on developing and propelling technological innovation to tackle global health challenges.

However, technological, business, and social sectors are highly influenced by the complexities of international relationships, national innovation systems, and the general national environment. Each can affect the discovery, development, effective implementation and equitable adoption of innovations which impact health (Matlin, 2008; McCannon et al., 2007). This illustrates the significance of an Integrated Innovation™ (scientific/technological, business, social innovation) approach to global health that aims to support the entire process of innovation from beginning to end, in light of these factors. Recently, there has been a major push to conceive of health innovation as a multi-sectoral process, reflected through institutional funding and training (e.g. University of Toronto Institute of Global Health Equity and Innovation, National Institutes of Health Framework Programs for Global Health Innovation) (University of Toronto, 2013; National Institutes of Health Fogarty International Centre, 2012), and nonprofit organizations (e.g. PATH) (PATH, 2013) and higher level policy discussions within European organizations (Battams et al., 2011). Young innovators in both high income countries (HICs) and low and middle income countries (LMICs), who aim to achieve equitable health outcomes through innovation, must receive the proper mentorship, training and support so that they are better able to navigate complexities and mitigate gaps in skills, knowledge or resources during the innovation process. A vast literature speaks to the need for and provides specific examples of mentorship, training and support for innovators in different health sectors – clinicians in academic medicine, public health, academic entrepreneurs, etc. (Manabe et al., 2009; Mahayosnand & Stigler, 1999; Jackson et al., 2003). Challenges faced by innovators are often focused on scientists failing to cross the innovation-to-commercialization gap (the “Valley of Death”) due to their lack of understanding of commercialization, entrepreneurship, product development, market value and general business concepts (Markham, 2002).

In the field of global health, young innovators are likely to face similar challenges, compounded by the complexities of innovation processes in different national innovation and public health systems. The Global Forum for Health Research aims to support strengthening of LMIC research and innovation systems for health, focusing on social, scientific/technological and business innovation research to impact on global health. The Canadian Coalition for Global Health Research (CCGHR), a network of global health researchers, organizations, and students, has expertise in mentorship, leadership and capacity development in global health research more generally. Grand Challenges Canada™ (GCC), funded by the government of Canada, is a non-profit organization that supports global health innovators in LMICs as well as Canada. However, we were unable to identify any sources that systematically examined requirements for and gaps in mentorship, training and support for global health innovators across the three Integrated Innovation™ sectors.

Hence, we conducted this scoping review to systematically scan the evidence for mentorship, training and support of innovators who would take an Integrated Innovation™ to global health innovation. We used GCC's organizational process and innovation funding cycle for global health innovators as a case example to which we could apply our review findings.

## 2. Methods

We chose a scoping review approach given our desire: (1) to map the extent, range and nature of research activity; (2) to help determine whether a full systematic review would be valuable and feasible; (3) to summarize and disseminate findings for an audience of innovators, mentors and funders; and (4) to identify research gaps in the current literature (Arksey & O’Malley, 2005).

**Figure 1:**
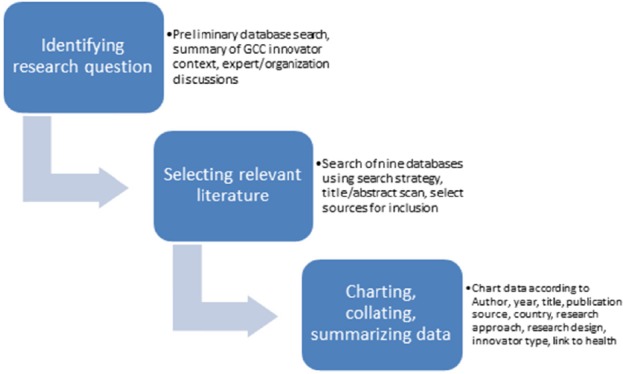
Methods schematic

Our review consisted of three phases between May and August 2012: (1) identifying the research questions, (2) selecting relevant literature and (3) charting, collating, summarizing and interpreting our findings (Figure 1).

### 2.1 Identifying Research Questions

To improve our review's applicability to our case of interest, we briefly summarized information on GCC Stars in Global Health Round 1 and 2 grantees (n=34).

As shown in Table 1, the 34 Stars in Global Health projects had been under way between 9-12 months. The grantees were relatively young in their careers as eligibility to participate in the first two rounds was restricted to those within ten years of attainment of their latest degree and most were from science/technological, academic backgrounds. Few mentors or mentees had business related experience, though a number had social innovation experience in partnerships (see http://www.grandchallenges.ca for further details).

**Table 1:** Grand Challenges Canada (GCC)™ stars in global health program

“Stars in Global Health” ideally demonstrate the coordination of scientific/technological, business and social innovation to propel a bold idea that will have an impact in global health. This approach, called Integrated Innovation™, recognizes that scientific/technological innovations for health are more likely to be scaled up and implemented with impact and sustainability, if they are developed with social and business innovations from the onset (Grand Challenges Canada, 2010). The program adopts an ‘innovation-push’ approach (Malinen et al., 2008) funding new investigators from Canada or LMICs to develop, modify and validate their innovation through Phase I proof of concept grants.
The majority of Phase I grantees in the first two rounds (n=19 in round 1, n=15 in round 2) were concentrated in science and academic research and affiliated with academic institutions. In round 1, two had experience in the corporate sector related to research and development and consulting and one mentioned formulating plans for commercialization links as part of the project. In round two, two of fifteen grantees had a business educational background (MBA) and five had some business/corporate experience in research and development consulting, partnering with joint ventures, director of program managing innovations, MBA background. Types of global health innovations have included: point of care diagnostic devices, the use of mobile health technologies to decrease maternal and child deaths, vaccine development and energy-efficient water purification systems.
Grantees identify a mentor on their project for support and guidance. Most Stars in Global Health grantees to date have been linked to mentors by background or expertise relevant to project components. Subsequently, these mentors should support grantees to pursue Phase II scale up grants by getting ‘buy-in’ from for-profit and not-for-profit partners to implement high quality and affordable innovations in alignment with local and regional social contexts in LMICs. For this stage, GCC offers online resources to help respond to the challenges that may arise when taking a global health innovation to scale and implementation.

The review team included experience in commercialization of science-based health innovations in LMICs, strategy and operations analysis, and coordination of the Stars in Global Health program, all from GCC, along with global health research capacity development in LMICs. An MPH student specializing in health promotion and global health led discussions of the grantee context and GCC needs to formulate three questions for exploration in this scoping review:

1) What is the nature of evidence around mentorship, training and support for innovators in global health?
2) How is the Integrated Innovation™ approach in global health being reflected in the mentorship, training and support for innovators? And
3) What type of initiatives in mentorship, training and support for innovators are applicable to the GCC Stars in Global Health program and implementable in its structure and context?

### 2.2 Identifying Relevant Papers

Preliminary literature searches helped formulate a search strategy that encompassed the main concepts, appropriate terms and the most relevant databases.

As seen in Table 2, keywords within each topic category were combined with OR. Across topic categories, terms were combined with AND. The search strategy was piloted and refined during consultations with an information specialist, tested in various databases to ensure that relevant results were appearing. Eight electronic databases, chosen for their multidisciplinary focus and relevance to the research questions, were systematically searched for their earliest available year to June 2012 (see Supplementary File Table 1 for details): Medline, BioMed Central, Business Source Premier, Embase, Scopus, Web of Science, Social Sciences Abstracts, and ProQuest.

**Table 2:** Keyword strategy used in searches

Topic Categories		Keywords
***Focus: Integrated Innovation***	Scientific/technological innovation	Biopharm* AND innova* AND health
		Biomed* AND innova* AND health
		Biotech* AND innova* AND health
		Research and development AND innova* AND health
		Information communication technolog* AND innova* AND health
		Medical innova*
		Point of care AND innova* AND health
		Technolog* AND innova* AND health
		Medic* AND innova* AND health
		Scienc* AND innova* AND health

	Business innovation	Entrepreneur* AND innova* AND health
		Business AND innova* AND health
		Commercializ* AND innova* AND health
		Technology transfer AND innova* AND health

	Social innovation	Diffusion of innovation AND health
		Ethic* AND innova* AND health
		Health care AND innova*
		Health system AND innova*
		Health delivery AND innova*
		Economic* AND innova* AND health
		Politic* AND innova* AND health
		Legal* AND innova* AND health
		Social* AND innova* AND health
		Cultur* AND innova* AND health
		Policy AND innova* AND health
		Determinants of health AND innova*
		Human resource* AND innova* AND health
		Leader* AND innova* AND health
		Social change AND innova* AND health
		Equity AND innova* AND health
		Govern* AND innova* AND health
		Implement* science AND health
		Translation* science AND health
		“Scaling up” AND health
		“Scale up” AND health
		“Scaling-up” AND health
		“Scale-up” AND health

***What: Training, support, mentorship***		Advisor
		Mentor*
		Mentee*
		Supervis*
		Teach*
		Support*
		Train*
		Coach*

***Who: Innovators***		Innovator*
		Researcher*
		Scientist*
		Clinician*
		Professional* (Lawyers, Vets)
		Practitioner*
		Health professionals (Physicians, Nurses, Naturopathic doctors, Chiropractors)
		Students (MPH, MBA)
		Investigators (PhD)

### 2.3 Paper Selection

Retrieved English titles and abstracts were independently reviewed by one team member for initial relevance related to innovation, health, and mentorship/support/training. A five percent random sub-sample was reviewed by a second team member. Raw inter-rater agreement on relevance was 77%. Formal inclusion and exclusion criteria were devised at this stage, and these two researchers independently reviewed each title and abstract for relevance, with resolution of disagreements by consensus. Major reasons for exclusion were: (1) no focus on health; (2) no focus on innovation; (3) no mention of type, potential, or need for support/training/mentorship of innovators. Included papers that were accessible by University of Toronto libraries were reviewed in full. Reference lists from papers included for full review were searched, and relevant papers to the research questions were also retrieved. Mendeley software was used for data management.

### 2.4 Mapping the Data

The following information was extracted from each paper: author(s), year, title, publication source, country, paper type, type of stakeholder(s), link to Integrated Innovation™ approach, and type of support/training/mentorship, either needs described or/and specific activities. The review leads held meetings to discuss extracted data and to synthesize our findings, following an inductive approach.

## 3. Results

The final search strategy generated 1535 titles and abstracts. Following initial title and abstract reviews and hand-searching of reference lists, 28 papers were judged relevant in this scoping review (full details in supplementary file).

### 3.1 What is the Nature of Evidence around Mentorship, Training and Support for Innovators in Global Health?

Most papers were published recently, with half (n=14) published in 2010. Eighteen (64%) were based in LMICs and the remainder in the USA and Canada (n=10). Most discussed all three of scientific/technology, business and, social innovation (n=23), with the rest (n=5) not including social innovation in a substantive manner. Four only discussed needs, ten focused on activities, half covered both. Nine provided perspectives (commentaries, editorials, etc.), four described programs of different kinds and 15 (56%) took some kind of empiric approach.

Eight of the latter papers included information on outcomes, including a promising framework for evaluation (Allen et al., 2010). Some reported students trained (Oden et al., 2010), university-industry collaborations initiated (Rezaie et al., 2008), companies developed (Chakma et al., 2010), and locally relevant technologies translated (Shah et al., 2010). One (Oden et al., 2010) reported a specific training outcome: 95% alumni commented that the program changed/modified their career plans to focus on global health medicine, research, and/or policy. Unfortunately, none provided sufficient evaluation data in comparative designs to determine the effectiveness of support, training, or mentorship activities in global health innovation.

### 3.2 How is an Integrated Innovation™ Approach in Global Health Being Reflected in the Mentorship, Training and Support for Innovators?

Drawing primarily on the empirical studies which included activities oriented by all three types of innovation (n=15) we found a wide variety of relevant activities, half with multiple stakeholders, reflecting innovation system perspectives.

As outlined in Table 3, the specific activities in LMICs to support, train and mentor innovators for health innovation aimed to fill identified needs included: business incubators, support organizations and centres for entrepreneurship, technology transfer and intellectual property management, internship programs for business skill development, government funding, and initiatives to bridge industry and researchers, and platforms for South-led innovation for global health. There was a heavy emphasis on supporting, training and mentorship of innovators in the science-business knowledge and resource gap. The focus was primarily on commercialization for product-based health innovation with little explicit mention of training to understand the social contexts in which innovations are to be implemented.

**Table 3:** Empirical papers relevant to mentoring, training and supporting global health innovators with an Integrated Innovation™ approach

First Author (date)	Country (ies)	Type (s) of stake-holder(s)	**Support/Training/Mentorship** (N=Needs, A=Activities, O=Outcomes)	
Al-Bader et al. (2009)	South Africa	Government	N
				Improving skills gap
				Training of scientists in business and entrepreneurial skills
				Mentorship from business community with health biotechnology experience
			A	Hellfire internship program to develop business and specialist skills
			O	Judged ‘successful’ but no longer operational
Al-Bader et al. (2010a)	Ghana, Rwanda, Tanzania, Uganda	Government, research institutes, university, private sector	N
				Platforms to enable access to financing
				Trust between stakeholder interactions
				Local insight, priorities and tacit knowledge
				Policies and strategies around innovation
			A	Cluster-building, business incubation
				Innovation platform to bring together science, business, capital partnerships and collaboration
Al-Bader et al. (2010b)	Ghana	Government, research institutes/Universities, private sector, NGOs and donors	N	Business knowledge in health sectors
				Links to industry and local stakeholders (i.e. traditional healers)
				Regulatory harmonization
				Intellectual property regulation
				R&D and health research spending
				Product development understanding
				Trust in stakeholder interactions
				Knowledge of local market
			A	Linking science and technology to health objectives
				Technology Consultancy Centre
				EMPRETEC entrepreneurship support organization
				National Board of Small Scale Industries
				GRATIS public agency for tech development and transfer
				TechnoServe entrepreneurship support organization
				Working group on health innovation
Allen et al. (2010)	Tanzania	Researcher observing private sector	A	Framework for health research in LMIC settings
				Combination of social sciences research methods and business model aspects of social entrepreneurship
			O	Evaluation of framework by examining achievement of milestones
Chakma et al. (2010)	South Africa	Government, private sector	N	“Soft services” such as hands-on networking and mentorship
				Pairing scientists with entrepreneurs
				Support from early to late-stage function of innovation development and commercialization
			A	Publicly funded virtual incubator to develop life science ventures
				Provide business advisory services, network contacts
				Serve as intermediary between granting agencies and investors
				Focus on mentoring early-stage entrepreneurs
				Hellfire internship program for young scientists to create a pipeline of scientist entrepreneurs for start-ups
			O	Development of companies, lessons learned
Frew et al. (2007)	India	Government, research institutes, private sector, NGOs and donors	N	Government policies and support, expertise of private sector for early-stage product development
				Targeted funding approach Improved public health infrastructure
				Incentives for private firms to develop innovative distribution strategies
			A	Recommended based on study:
				Single agency to provide science mentoring
				Local collaborations between R&D and research institutions
				Access to government-sponsored research funds
				Focus on developing innovations to address local health needs
				New Millennium Indian Technology Leadership Initiative Program
Frew et al. (2008)	China	Government, research institutes, private sector, NGOs and donors	N	Individuals understanding science and international regulations/intellectual property protection, manufacturing and product registration
				Political will, training programs, international collaborations, creation of biotech industrial parks
				Training students in industrial setting
			A	High-tech R&D ‘863 program’ focused on applied research and commercialization
Kamunyori et al. (2010)	Uganda	Government, research institutes, private sector, NGOs and donors	N	Expertise in drug regulation
				Consolidated innovation policy, ministry
				Linking innovation initiatives effectively
				“Demand driven” training programs
			A	Millennium Science Initiative (MSI): Window C – private sector cooperation bridging industry-research divide through monetary incentives for collaboration
				Presidential Support to Scientists Fund: government funding support for commercialization
				Ugandan Industrial Research Institute (UIRI) incubator program
				Makerere University IP management policy, technology transfer office
				Innovations at Makerere program
				Makerere University Private Sector Forum to match industry need with university research and training
				University curriculum to meet private sector skills need
				Programs to encourage links between industry and research
				Uganda National Academy of Sciences – pairing MPs and Ugandan scientists
			O	MSI has not achieved intended level of collaboration
Nwaka, S. et al. (2010)	Africa	Funder	A	Partner, fund, coordinate innovation from discovery to manufacturing of drugs, diagnostics, vaccines, medical devices Links health and innovation to economic development
Oden et al. (2010)	US	University	A	Project course to develop solutions for global health challenges provided by clinical partners
				Pairing of project teams with local mentors to guide and evaluate design process
				Opportunity to go and implement solutions that have been designed
				Course development for MBA and senior-level engineering students; field research to write business plans for global health technologies
			O	40 technologies and educational program designed by 333 students since 2006; 28 implemented in sub-Saharan Africa, Latin America, Carribean, Southeast Asia, US; 18,000+ have benefited; 95% alumni commented that the program changed/modified their career plans to focus on global health medicine, research, and/or policy
Perampaladas et al. (2010)	Nigeria	Government, research institutes, private sector, NGOs and donors	N	Support business-friendly environments
				Benefit-sharing agreements for equitable partnerships between scientists and local traditional healers
				Quality control in training
				Applied training programs, mentoring and internships on-the-job
				Fostering of partnerships to fill gaps in knowledge and technical expertise
				Bio-entrepreneur champions to manage partnerships, recruit professionals, link with government holding dual scientific and business roles
Rezaie et al. (2008)	Brazil	Government, research institutes, private sector, NGOs and donors	N	Expansion of academic and executive programs in entrepreneurial training for health biotech sector
			A	State University of Campinas – innovation and technology transfer activities
				Partnerships for Technological Innovation program
			O	Encouraged increased number in university-industry collaboration but effectiveness unclear
Shah et al. (2010)	Tanzania	Government, research institutes, private sector, NGOs and donors	N	Investment in infrastructure to support innovation in government policies
				Funding for commercialization of innovation and venture capital
				Awareness for intellectual property protection
				Strong enforcement systems for patents
				Capturing local products with commercial value
				Successful linkages with private sector
				Environment conducive for private sector investment
				Shared values, entrepreneurial spirit, capital for collaboration between business and science
				Innovation platform mechanism
			A	Government ministries, committees to coordinate science, technology and innovation, regulation authorities
				Technology transfer policies and offices
				Entrepreneurial leadership initiatives at universities and research institutes; “Clubs, Clusters and Incubators” program
				Signing of MoU with private sector company
				Infrastructure and partnerships in research institutes and universities for biotechnology
				National business plan competitions
				Capacity strengthening activities in research
			O	Success in development and translation of some locally relevant technologies, entrepreneur training, building links between public and private partners
Simiyu et al. (2010a)	Kenya	Research institutes	N	Innovation management
				Institutional intellectual property policies
				Linkages with investors
				Technology assessment
			A	Training programs offered by Bridgeworks Africa (local venture capital firm)
			O	Development of institutional IP policy, establishment of relationships and training programs at international organizations
Simiyu et al. (2010b)	Rwanda	Government, research institutes, private sector, NGOs and donors	N	Government policies on innovation and innovation management
				Links within private sector and researchers, venues for knowledge and idea exchange
				Scientific infrastructure and skilled scientists
				Support of innovative activities through funding
				Coordination of R&D and innovation
			A	Innovation and technology transfer units
				Seminars on intellectual property management
				Centre for Innovation and Technology Transfer to develop appropriate solutions for rural areas
				Incubation facility
				Regional centres at Rwanda Private Sector Foundation to train for small business management skills
				Science and Technology in Education work plan
				Government funds to support innovation activities
				Online courses on scientific areas as well as entrepreneurship, rewards for scientists, development of IP policies

## 4. Discussion

Given the newness of the global health innovation field, our mapping found papers on perspectives, new programs, and a range of activities to support global health innovators but limited empirical work to inform training, mentoring and support practice. Literature on mentorship in global health clinical research (Shah et al., 2011) and academic medicine (Nelson et al., 2012) are focused on helping trainees achieve research productivity, career development or appropriate conduct in global settings. These important objectives of mentorship need to be complemented for global health innovators by helping trainees understand the cycle of innovation and the social contexts in which innovations will be discovered, developed, scaled up, deployed, and sustained.

### 4.1 System Level

Papers identified in this scoping review did indicate a number of mechanisms by which support, training and mentorship of global health innovators may be facilitated with the involvement of micro to macro level stakeholders. In LMICs, the identified mentorship, training, and support structures were highly focused on capacity building within the innovation system itself so that innovation might be better facilitated. A good example of a program (unfortunately without evaluation data) is the African Network for Drugs and Diagnostics (Mboya-Okeyo, Ridley, & Nwaka, 2009). It enables African-led research and innovation for discovery, development and delivery to treat diseases affecting Africa (science/technology), with a focus on translation of innovations (social) and also aims to attract commercial investments and collaborations between South-South and North-South partners (business).

### 4.2 Institution Level

Responding to Hotez and colleagues’ (2005) call for public health institutions to train students in appropriate technology, and to ensure core competencies are reflective of technical and “real world” skills for product development and use, is Northwestern University's tripartite collaborative model (Palamountain et al., 2010) in which diagnostic companies donate health technology innovations, and students from schools of engineering, business, medicine and social science adapt and market them for LMIC settings. Oden and colleagues (2010) provided initial empiric data on the role of university-based training effort which modified students’ future career plans.

### 4.3 Role of Funders

Beyond the funding primarily for global health innovation activities (Nwaka et al., 2010), funders of innovators training in an Integrated Innovation™ approach have a range of opportunities. The National Institutes of Health Fogarty International Center's Framework Programs for Global Health Innovation is one example (National Institutes of Health Fogarty International Center, 2012). This program supports US and LMIC institutions to develop interdisciplinary, postdoctoral training programs for global health innovation products, processes and policies. Funder- sponsored opportunities offered to grantees have included: online websites and web sources to foster collaboration, providing opportunities to discuss with previous grantees, independent consultation, workshops, trainee development programs and curricula, establishing partnerships between industry, government, non-government, academic organizations, virtual forums for collaborations, and “knowledge broker” approaches.

### 4.4 Application to GCC Stars in Global Health Program

**Figure 2:**
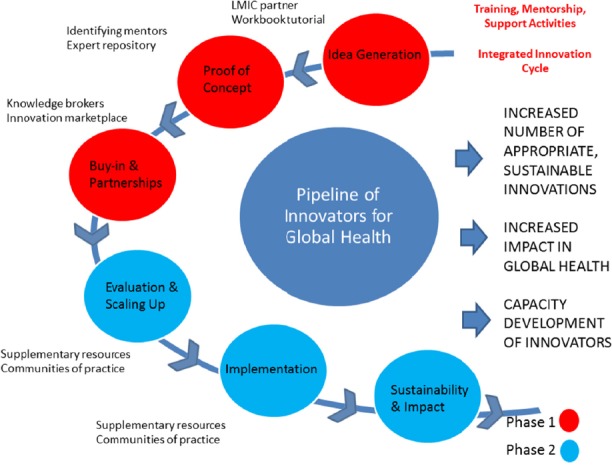
Pipeline of innovators for global health

The approaches we uncovered in our review may apply at different stages of the innovation process, enabling discovery, development, implementation and sustainability of innovations for greater impact on global health, Mentoring, training and support could be conceptualized holistically from start to finish throughout the process of global health innovation. We applied approaches uncovered in our review to the phases of GCC's innovation cycle (see Figure 2) which build on GCC's current online proposal development resource, that is updated frequently with relevant content, and its LinkedIn group to facilitate collaboration.

In Phase I, GCC (and other global health innovation funders) could focus on partnerships and skill-building whereby grantees ground themselves in how their projects should be defined and conceptualized through an Integrated Innovation™ approach. Partnering to identify LMIC needs, context and partnerships and identifying other mentors who are not in the same field of expertise as the innovator, may fill gaps when undertaking proof of concept studies. Milestones in reporting for mentorship activities could be elicited. A repository of experts, particularly with business and social innovation acumen (as these areas of expertise seem to be lacking the most) and who have experience in global health contexts, could be built for grantees to contact if needed. Knowledge brokers could act as “middlemen” to guide and mentor grantees on bridging the challenges in taking an innovation to a specific commercial market, as well as to help with building core skills (e.g. writing a business plan). Innovations that merit Phase II funding could be showcased as part of an innovation marketplace for potential partners and investors, which would help grantees gain skills in presenting, making elevator pitches, networking, etc.

In Phase II, supplementary sources addressing implementation science, translational science, product development partnerships (Rabin et al., 2008; Yamey, 2011) could be added to the online proposal development resource, which would supplement the entrepreneurship skillsets being built to help fill in knowledge and practice gaps around evaluation, scaling up, and implementation. Communities of practice, which have been utilized widely in business and health sectors (Li et al., 2009), could be facilitated amongst these innovators to share tacit knowledge and learning. The partnerships and networks built through interactions amongst the innovators, potential partners and investors, as well as the knowledge and skills gained through the training, mentorship and support activities could enable capacity development of innovators as they move to achieve impacts on coverage and equity.

### 4.5 Limitations

As only papers written in English were included, other sources that may have been relevant may have been missed. In addition, due to financial resources and time, only papers that were available through the University of Toronto libraries were included. Nevertheless, the scoping review was sufficient to indicate the state of evidence available.

## 5. Conclusions

Our scoping review has identified the nature of literature on global health innovators and their organizations, funders, and educators relevant to training, mentorship and support of innovation in global health. It applied our findings to one programme aiming for Integrated Innovation™ in GCC's Stars in Global Health. More rigorous research and evaluation is needed to determine better processes for programmes to train, mentor and support innovators in global health and their effectiveness in impacting on global health equity.
